# Neural mechanisms of rhythm-based temporal prediction: Delta phase-locking reflects temporal predictability but not rhythmic entrainment

**DOI:** 10.1371/journal.pbio.2001665

**Published:** 2017-02-10

**Authors:** Assaf Breska, Leon Y. Deouell

**Affiliations:** 1 Department of Psychology, Hebrew University, Jerusalem, Israel; 2 Edmond and Lily Safra Center for Brain Sciences, Hebrew University, Jerusalem, Israel; New York University, United States of America

## Abstract

Predicting the timing of upcoming events enables efficient resource allocation and action preparation. Rhythmic streams, such as music, speech, and biological motion, constitute a pervasive source for temporal predictions. Widely accepted entrainment theories postulate that rhythm-based predictions are mediated by synchronizing low-frequency neural oscillations to the rhythm, as indicated by increased phase concentration (PC) of low-frequency neural activity for rhythmic compared to random streams. However, we show here that PC enhancement in scalp recordings is not specific to rhythms but is observed to the same extent in less periodic streams if they enable memory-based prediction. This is inconsistent with the predictions of a computational entrainment model of stronger PC for rhythmic streams. Anticipatory change in alpha activity and facilitation of electroencephalogram (EEG) manifestations of response selection are also comparable between rhythm- and memory-based predictions. However, rhythmic sequences uniquely result in obligatory depression of preparation-related premotor brain activity when an on-beat event is omitted, even when it is strategically beneficial to maintain preparation, leading to larger behavioral costs for violation of prediction. Thus, while our findings undermine the validity of PC as a sign of rhythmic entrainment, they constitute the first electrophysiological dissociation, to our knowledge, between mechanisms of rhythmic predictions and of memory-based predictions: the former obligatorily lead to resonance-like preparation patterns (that are in line with entrainment), while the latter allow flexible resource allocation in time regardless of periodicity in the input. Taken together, they delineate the neural mechanisms of three distinct modes of preparation: continuous vigilance, interval-timing-based prediction and rhythm-based prediction.

## Author summary

Making predictions is a major adaptive brain function. Predicting the timing of upcoming events enables the brain to prepare for them. However, it is unclear how this is achieved. It is believed that in rhythmic environmental context, such as in music and speech, temporal predictions are achieved by synchronizing the naturally occurring brain rhythms to the external rhythms, a process referred to as oscillatory entrainment. We tested whether rhythm-based predictions have unique expressions in behavior and brain activity—as measured by electroencephalogram (EEG)—by comparing rhythmic streams to less rhythmic streams that enable prediction based on memorizing repeating intervals. We show that phase concentration (PC) of neural oscillations—a neural pattern commonly associated with entrainment—occurs to a similar extent in both predictive rhythmic and less rhythmic streams. Using a computational model, we show that these results are not consistent with the predictions of an entrainment mechanism, indicating that PC is not a unique indicator of entrainment. However, we also show that when an expected event is omitted, memory-based predictions can be flexibly reoriented, while rhythm-based predictions lead to immediate obligatory resource withdrawal and larger behavioral costs. This is consistent with resonance, a different prediction of entrainment model. As a whole, the results identify the overlapping and distinct mechanisms of rhythm- and memory-based temporal predictions.

## Introduction

Information processing is optimized by predicting not only the location or identity of upcoming events but also their timing and allocating resources accordingly [1–6]. Understanding the mechanisms of temporal prediction is thus central for models of cognitive and neural processing. One ubiquitous source for temporal predictions is regularity in the stimulus stream, mainly when events appear rhythmically, as in speech, music, or biological motion [3,4,6–9]. It has been suggested that in such cases, a costly “vigilance mode” is replaced by a metabolically efficient “rhythmic mode” by entraining low-frequency neural oscillations to the external rhythm [10,11]. In this study, we tease apart the neural manifestations of oscillatory entrainment from rhythm-independent mechanisms of temporal predictions. This is done by comparing temporal prediction based on rhythms to predictions based on memory, a critical comparison that was so far missing.

Neural excitability fluctuates in low frequencies from sub-delta to alpha [12,13], with apparent optimal phases for performance in these frequencies, as indicated by findings of an association between phase and performance [3,7,14,15]. According to entrainment models, such endogenous oscillations can be entrained (i.e., undergo period correction and phase alignment) to an external rhythmic stream such that optimal phases align with on-beat times of rhythmic stimuli. Consistent with this idea, behavioral performance is facilitated for rhythm on-beat compared to off-beat times [9,16–20]. In addition, electrophysiological recordings found that forming temporal predictions based on rhythmic streams is associated with phase concentration (PC) of delta-band activity in task-relevant sensory regions [6,21,22], such that the optimal phase for performance is observed at the time of rhythmic stimuli [7] (for a review see [23]). This pattern is consistent with entrainment and is often treated as a direct indicator for this mechanism (e.g., [3,4,7,21,22,24]. Arguably, entrainment should be most applicable and efficient when the sequence is periodic. Conversely, entrainment should become less applicable and less efficient as the stimulus stream becomes less periodic (i.e., the stream intervals are more jittered), because stable alignment of endogenous oscillations with the stream is not possible.

However, temporal predictions can also be formed without periodicity, or any continuous stimulus stream, when the interval between an expected event and a preceding event is known based on previous experience [1,25,26] (for a review see [27]). Such predictions presumably operate through dedicated interval-timing circuits [28,29], forming temporal memory traces to which new intervals are compared [30,31]. This mechanism can operate regardless of the periodicity of the stimulus stream as long as there is knowledge regarding the temporal contingency. This raises the question whether temporal predictions in rhythmic scenarios are mediated by oscillatory entrainment, by “memory-based” mechanisms based on the repeated exposure to the interval [32], or by both. Recent findings in a speeded discrimination task suggest that while any type of temporal prediction improves perceptual accuracy, only rhythms facilitate response speed, seemingly in line with the latter alternative [33]. However, other studies found that response speed can also be facilitated by memory-based predictions [25,26,34].

To date, no study has directly compared the neural expressions of these two prediction modes, namely, memory-based and rhythm\entrainment-based. Electrophysiological studies have associated rhythm-based predictions with phase adjustments of delta-band activity [3,6,7,21,22] and of the envelope of alpha activity (amplitude reduction prior to on-beat times [34,35]). However, as these were not tested in memory-based predictions, it is impossible to determine that they are unique to rhythm entrainment. Rhythm-based predictions are also associated with the contingent negative variation (CNV) potential peaking at on-beat times and resolve immediately after [9,34] and with earlier latency of the P3 potential [8,36]. However, separate studies found CNV and P3 effects for memory-based predictions as well [26,37,38], and in the absence of direct comparison, it is impossible to determine the unique contribution of rhythmicity to these effects.

In the current study, we identify the unique mechanisms of rhythm-based processing by directly comparing the behavioral and electroencephalogram (EEG) expressions of temporal predictions in a perfect periodic condition and in two less periodic conditions, one that enabled formation of memory-based temporal predictions and one that provided no temporal information for prediction. We show that the patterns of delta-band PC, and also of alpha-band envelope and target-evoked event-related potentials (ERPs), are identical in the rhythmic- and memory-based conditions. We also show that the delta PC pattern is inconsistent with a computational model of oscillatory entrainment, which predicts weaker entrainment for the less periodic stimulation of the memory-based condition. However, we also find evidence for rhythm-unique neural patterns: violation of predictions results in immediate obligatory resource withdrawal and larger behavioral costs when rhythm-based, while memory-based predictions are more flexible, in line with the concept of resonance that is predicted by oscillatory entrainment.

## Results

### Behavior

**Fig 1:**
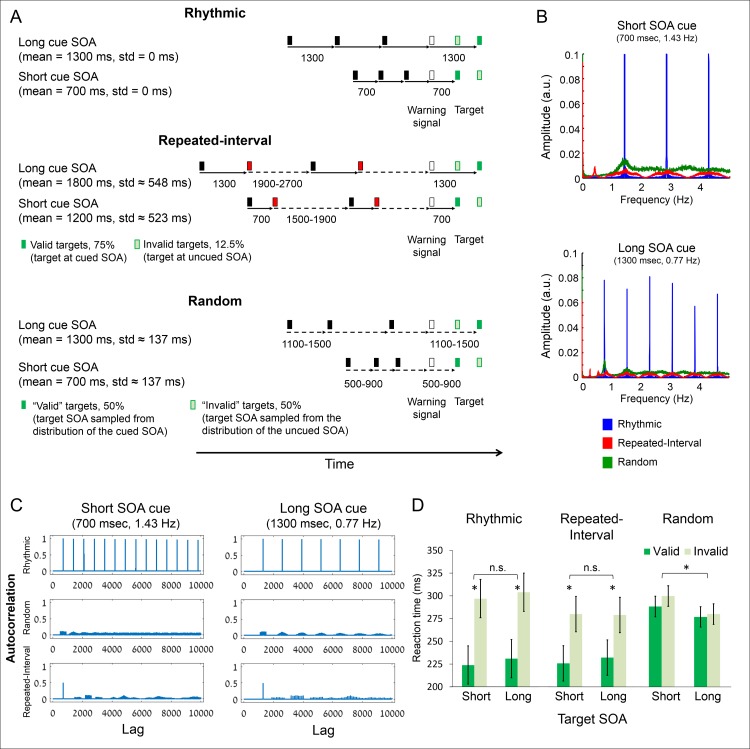
Experimental paradigm and behavioral results demonstrating larger cost but not benefit for rhythm-based temporal predictions. (A) Subjects detected targets embedded in a stream of visual stimuli. In the Rhythmic condition, the stream intervals were fixed. In the Repeated-Interval condition, every black-to-red interval was fixed and red-to-black jittered. In the Random condition, all intervals were jittered around the fixed interval. In the first two conditions, the target (dark green) appeared at the fixed SOA relative to a WS (white) in 75% and in the other SOA in 12.5% of the trials (light green with dark green edge). The remaining 12.5% were catch trials in which no target appeared, to prevent anticipatory responses to long SOA targets. In the Random condition, the target SOA was drawn from the same distribution as the stream SOA in 43.75% of the trials and from the other distribution in the other 43.75%, and 12.5% of the trials were catch trials. (B) Spectral representations of the stimulus sequence in the three experimental conditions. The stimulus sequence in each experimental condition was modeled as a time series that contains one at stimulus onset and zero otherwise (all trials were concatenated). The amplitude at the cued frequency as well as in delta frequencies (0.5–3 Hz) is strongest in the Rhythmic condition, weaker in the Random condition, and weakest in the Repeated-Interval condition. (C) Autocorrelation functions of the stimulus sequence in the three experimental conditions, calculated on the same modeled stimulus sequences that were used to create the spectral representations in B. The Rhythmic condition has high autocorrelation in fixed lags because of the perfect periodicity; the other conditions have relatively low autocorrelation at varying lags. (D) Mean reaction times in each combination of SOA and cue validity in the three experimental conditions. Error bars represent standard errors of the validity effect within each SOA and condition. *p < 0.05.

**Fig 2:**
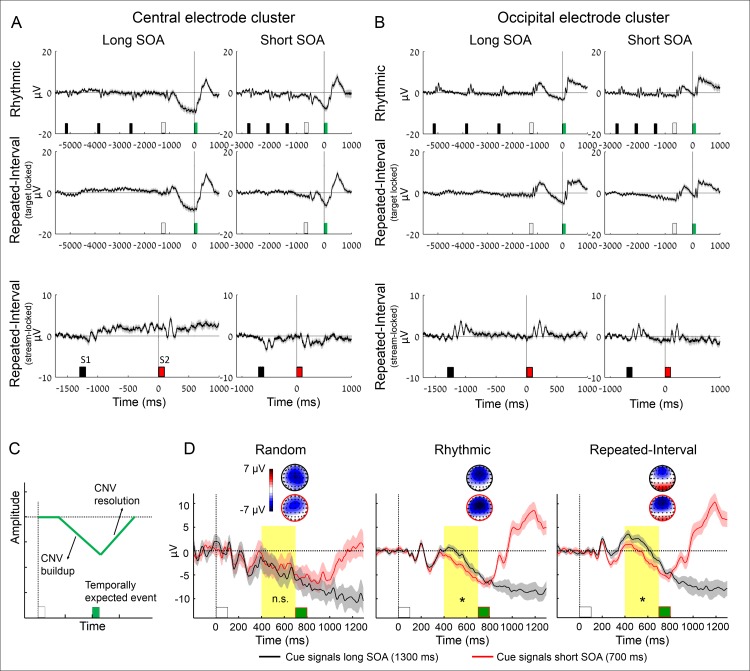
The CNV buildup is similarly modulated by rhythm- and memory-based temporal predictions. (A, B) Group-averaged ERPs elicited by the stimulus sequence in the different conditions in central (A) and occipital (B) electrode clusters. Black/red bars: sequence stimuli (prior to the WS), white bars: WS, green bars: targets. Top row: Rhythmic condition, locked to the WS, in an interval extending from the third stimulus prior to the WS up to the target (all trials had at least three stimuli prior to the WS). Middle row: Repeated-Interval condition, locked to the WS (interval identical to the Rhythmic condition). Bottom row: response to the last pair preceding the WS-target pair in the Repeated-Interval condition, locked to the first stimulus of the pair (S1). These pairs are not presented in the middle row, as their timing was jittered relative to the WS-target pair. (C) Schematic illustration of the CNV stages. (D) Group averaged responses (average across a 9-electrode central cluster) locked to the WS (white bar) onset. Note more negative CNV waveforms when expecting a target at the short (red) compared to the long SOA (black) in the two predictive conditions. Error margins reflect the standard error for the difference between SOAs; scalp topographies are averaged across 650–700 ms post-WS, just before the short SOA target (green bar). Yellow background marks the predefined interval for analysis (*p < 0.05).

To test the unique signatures of rhythm-based predictions, participants (*n* = 21) responded to targets that followed a sequence of visual stimuli and a warning signal (WS, Fig 1A) in three different conditions. In the Rhythmic condition, the sequence was perfectly periodic (i.e., the sequence stimuli appeared with identical stimulus onset asynchrony [SOA]), and the target following the WS had high probability to appear on-beat, making its timing predictable. In the Repeated-Interval condition, every odd interval was identical to the SOA of the Rhythmic condition, whereas even intervals were jittered around a different interval duration and were never identical to the rhythm interval or an integer multiple of it. To equate the information provided by the Rhythmic and Repeated-Interval conditions, the number of repeated fixed SOA intervals in the Repeated-Interval was equal to the number of overall intervals in the Rhythmic condition. The target had high probability to follow the WS at the interval of the fixed SOA, making its timing predictable. In the Random condition, the sequence SOAs were jittered around the SOA of the Rhythmic condition, and the target SOA (interval between the WS onset and the target onset) was drawn from a wider distribution that was not centered on the rhythm SOA orthogonally to the sequence mean SOA. Thus, the sequence provided no information regarding the timing of the upcoming target. The Random and Repeated-Interval conditions were, therefore, much less periodic than the Rhythmic condition, as is indicated by (1) SOA variance: zero in the Rhythmic conditions, larger in the other conditions (Fig 1A); (2) frequency domain representation: strong amplitudes in the delta frequency in the Rhythmic condition (its base frequency and harmonics), lower amplitude in the other conditions (Fig 1B); and (3) autocorrelation function: high autocorrelation in the Rhythmic condition at equal lags, minimal autocorrelation in the other conditions (Fig 1C). We surmised that because of this variability, low-frequency oscillations would be less efficiently entrained to the stimulus stream in the Random and Repeated-Interval conditions. However, unlike the Random condition, the Repeated-Interval condition enables accurate temporal prediction because of the repeated presentation of the WS-Target SOA. Thus, any effects that are not different between the Rhythmic and Repeated-Interval conditions reflect temporal predictability, which is not driven by periodicity in the stream.

Our design allowed us to separately test two indicators of forming temporal predictions, namely, benefit and cost, using planned contrasts. First, we could examine whether having a context affording temporal predictions leads to a benefit relative to an unpredictable context and whether the benefit is different between rhythm- and memory-based predictions. For this purpose, we compared response times (RTs) of valid trials, in which the temporal prediction was fulfilled (i.e., targets appeared at the expected time), between the three experimental conditions (for analysis purposes, “valid” trials were defined in the Random condition as those in which cue and target SOAs were drawn from the same distributions; see Materials and methods for more details). Second, we could examine whether violating a temporal prediction leads to a cost relative to an unpredictable context and whether the cost is different between rhythm- and memory-based predictions. For this purpose, we compared RTs of invalid trials, in which targets appeared at an unexpected time, between the three experimental conditions.

RTs to targets were first analyzed using an omnibus three-way ANOVA with factors condition X target SOA X validity. RTs were overall shorter to valid compared to invalid trials (*F*_(1,20)_ = 76.37, *p* = 2.9 × 10^−8^, *ω*^*2*^_*p*_ = 0.64, Fig 1D), but the validity effect differed across conditions (interaction *F*_(2,40)_ = 18.69, *p* = 1.9 × 10^−6^, *ω*^*2*^_*p*_ = 0.22). For valid trials, RTs were faster relative to the Random condition both in the Rhythmic (*t*_(20)_ = 5.88, *p* = 9 × 10^−6^, C*ohen’s d* = 1.28) and the Repeated-Interval (*t*_(20)_ = 5.8, *p* = 1 × 10^−6^, *d* = 1.27) conditions with no difference between the two (*t*_(20)_ = 0.12, uncorrected *p* = 0.91, 95% confidence interval [CI] for the difference −6.23 to 6.99 ms). Thus, while both rhythm-based and memory-based predictions lead to a benefit relative to no prediction, there is no advantage to one of them over the other. Unlike for valid trials, responses to invalid trials were slower in the Rhythmic relative to the Repeated-Interval condition, (*t*_(20)_ = 3.14, *p* = 0.005, *d* = 0.69). Responses were also slower in the Rhythmic relative to the Random condition (*t*_(20)_ = 1.88, *p* = 0.037, one-tailed, *d* = 0.41), while the Repeated-Interval condition did not differ from the Random condition (*t*_(20)_ = 1.16, *p* = 0.26). Thus, unlike their similar benefits, the cost of violation of rhythm-based prediction was larger than that of memory-based predictions. Finally, there was a main effect of condition (*F*_(2,40)_ = 13.01, *p* = 4.4 × 10^−5^, *ω*^*2*^_*p*_ = 0.28) and a condition X SOA interaction (*F*_(2,40)_ = 6.5, *p* = 0.0036, *ω*^*2*^_*p*_ = 0.08). This was because of faster responses to long than short SOA targets (foreperiod effect, [27]) only in the Random condition (*F*_(1,20)_ = 8.04, *p* = 0.01, *ω*^*2*^_*p*_ = 0.14).

### Neurophysiology

#### Preparatory ERPs

The data of two participants were rejected from all EEG analyses because of excessive artifacts, leaving 19 datasets. Analysis of ERPs during the presentation of the sequence was focused on the CNV, a negative deflection that builds up over fronto-central sites in the interval between a WS and an impending event and resolves (towards baseline) when the latter has occurred [39,40]. We first examined the predictive conditions to test whether a CNV is observed during the presentation of the stream prior to the WS, only after WS, or in both time ranges. If the CNV reflects oscillatory entrainment, it could be expected to be elicited repeatedly in the Rhythmic condition during the entire stream. We observed a typical CNV after the WS in both the Rhythmic and Repeated-interval conditions (Fig 2A, top and middle rows, respectively) but no repeating CNV or any oscillatory pattern prior to the WS in the Rhythmic condition or the Repeated-Interval condition. Furthermore, no CNV was observed when locking to the final pair prior to the WS in the Repeated-Interval condition (Fig 2A, bottom row), confirming that the lack of CNV was not due to averaging across the temporally jittered stimuli. We repeated this analysis in an occipital electrode cluster to test for similar modulations or oscillatory pattern at the sensory level. Notably, no oscillatory pattern or any repeating deflection was observed in occipital electrodes during any of the conditions (Fig 2B). Instead, there were typical stimulus-evoked visual responses and a positive deflection following the WS that changed trajectory to a buildup of negativity till the time of the target. Importantly, locking to the final pair prior to the WS in the Repeated-Interval condition revealed no trace of a slow oscillation elicited by the S1 or S2 stimuli of the sequence (Fig 2B, bottom row). Thus, the CNV was not driven automatically by the rhythm when subjects were exposed to the stream and used it to form a temporal prediction but was only elicited when preparing for the target.

We compared two parameters of the CNV between the two sources of prediction: the rate of CNV buildup and its resolution after omission of a temporally expected event (Fig 2C). First, the CNV characteristically builds up faster as the expected interval is shorter [9,34,38]. We tested whether rhythm-based temporal prediction is associated with a distinct modulation pattern of the CNV buildup compared to the modulation that is associated with memory-based temporal prediction. To quantify this effect, we averaged the CNV amplitude in a time window preceding the short SOA target. We expected to find more negative waveforms in this window when the target was expected in the short compared to long SOA both in the Rhythmic and Repeated-Interval conditions and compared this effect between conditions. We found more negative amplitude of the CNV buildup when expecting the target at short compared to long SOAs in the Rhythmic (*t*_(18)_ = 2.81, *p* = 0.012, *d* = 0.64) condition as well as in the Repeated-Interval (*t*_(18)_ = 2.81, *p* = 0.012, *d* = 0.64) condition. Importantly, there was no difference between the effects of the expected SOA in the two conditions (interaction between the effects of SOA and condition: *F*_(1,18)_ = 0.18, *p* = 0.68, 95% CI for the difference −1.46 to 2.19 μV, Fig 2D). For illustration, we also present the Random condition, in which there was no temporal prediction, and, indeed, the SOA had no significant effect (*t*_(18)_ = 0.36, *p* = 0.72). Thus, the modulation of CNV buildup was affected by temporal predictions but did not show a rhythm-specific pattern.

**Fig 3:**
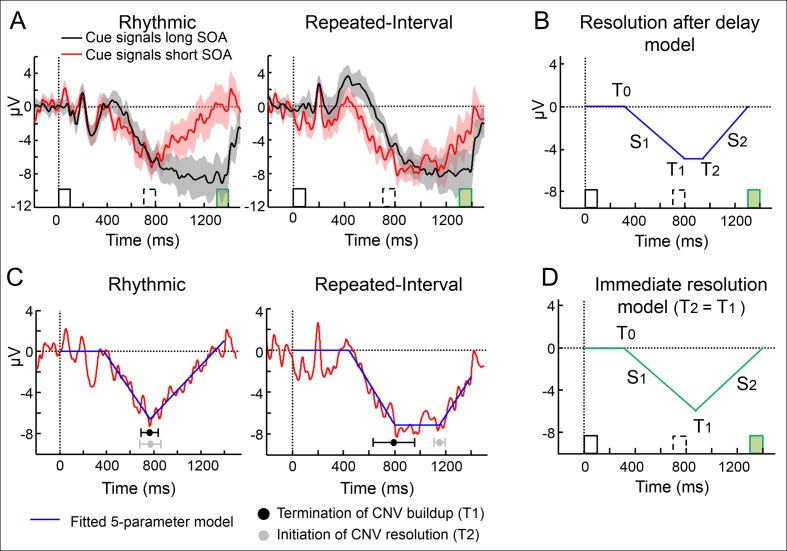
Immediate CNV resolution after omission of an expected event for rhythm-based temporal predictions. (A) Group averaged waveforms (central cluster electrodes) in the two predictive conditions exclusively for trials with long SOA targets (green bar). Black line—expecting the target in the long SOA; Red line—expecting it in the short SOA, but it is omitted (invalid short cue trials). Error margins—standard error for the difference between SOAs. (B) A five-parameter model that allowed a delay between buildup and resolution was fitted to the CNV waveform. (C) Waveforms of the Rhythmic and Repeated-Interval when an expected short SOA target is omitted (red) fitted with the five-parameter model (blue), with estimated latencies of termination of the CNV buildup and initiation of the CNV resolution. (D) A four-parameter model that coerced immediate resolution after buildup termination was used as a null model for model comparison.

Another characteristic of the CNV is that it resolves when an expected interval elapses even when the impending event is delayed [9,37,40]. In our data, this occurred in the Rhythmic and Repeated-Interval conditions when a target was expected at the short SOA but omitted (i.e., it unexpectedly appeared in the long SOA; Fig 3A). We examined whether the CNV resolution is different, in magnitude or latency, when the temporal prediction is based on rhythm compared to when it is based on memory. To quantify the CNV resolution, we fitted the CNV waveform (averaged across subjects) in the Rhythmic and Repeated-Interval conditions with a continuous model that included the latencies of buildup initiation, slope, and termination, and resolution initiation and slope as free parameters (Fig 3B). This approach is superior to quantifying the CNV resolution in a predefined interval [9] or using fixed length bins [37], which could miss the actual latencies of different CNV stages and distort the estimated slope if the trajectory is not linear during the entire interval.

Comparison of the estimated parameters between the two predictive conditions revealed that the time of initiation (T_0_), slope (S_1_), and termination (T_1_) of the CNV buildup and the slope of its resolution (S_2_) were not different between the Rhythmic and Repeated-Interval conditions (*t*_(18)_ = 0.34, 0.37, 0.03, 1.62, respectively, all *p* > 0.05). However, the start point of the CNV's return to baseline (T_2_) occurred later in the Repeated-Interval (1,152 ms) than in the Rhythmic condition (767 ms, *t*_(18)_ = 3.73, *p* = 0.0015, *d* = 0.86, Fig 3E and 3F). Furthermore, there was a significant gap between T_1_ and T_2_ in the Repeated-Interval condition (*t*_(18)_ = 2.17, *p* = 0.043, *d* = 0.5) but not in the Rhythmic condition (*t*_(18)_ = 0.09, *p* = 0.93). To justify the use of this five-parameter model, we took a model-selection approach (using the Bayesian Information Criterion, BIC) to examine whether this model is preferred to an alternative, less elaborate model in which the CNV resolution was immediate. That is, this four-parameter model did not include a free parameter for a delay between the termination of the CNV buildup and the initiation of its resolution. For each condition, the model with the lower BIC value was considered the preferred model. In the Repeated-Interval condition, the five-parameter “resolution after delay” model turned out to be superior over an alternative four-parameter “immediate resolution” model (Fig 3D) with no delay (ΔBIC = 76). However, in the Rhythmic condition, the four-parameter model was superior (ΔBIC = 7.3). This indicates that the five-parameter model is indeed necessary, as it is preferred for the Repeated-Interval condition but also provides converging evidence to the difference between conditions, as it was not preferred for the Rhythmic condition. Thus, in rhythmic streams, target omission triggered an immediate resolution of the CNV, while in memory-based temporal predictions, the initiation of the CNV resolution was delayed, presumably anticipating the upcoming event at the long SOA.

#### Alpha-band amplitude

Previous studies found that the amplitude of alpha-band oscillations (8–13 Hz) is reduced just prior to, or at the time of, an event that is temporally expected based on rhythms [9,34,35]. We tested whether this modulation of alpha-band amplitude is unique to rhythms or whether it is expressed to the same extent when the prediction is based on a memorized interval. To dissociate the effect of expectation from response to the target we analyzed the alpha-band amplitude in the time window of the short SOA (700–900 ms after the WS) exclusively in trials in which the target appeared at the long SOA. In this window, we expected to find in the Rhythmic condition lower alpha amplitude when expecting the target at the short compared to long SOA. If this phenomenon is unique to rhythm-based predictions, the Repeated-Interval condition should have smaller, or even no modulation.

**Fig 4:**
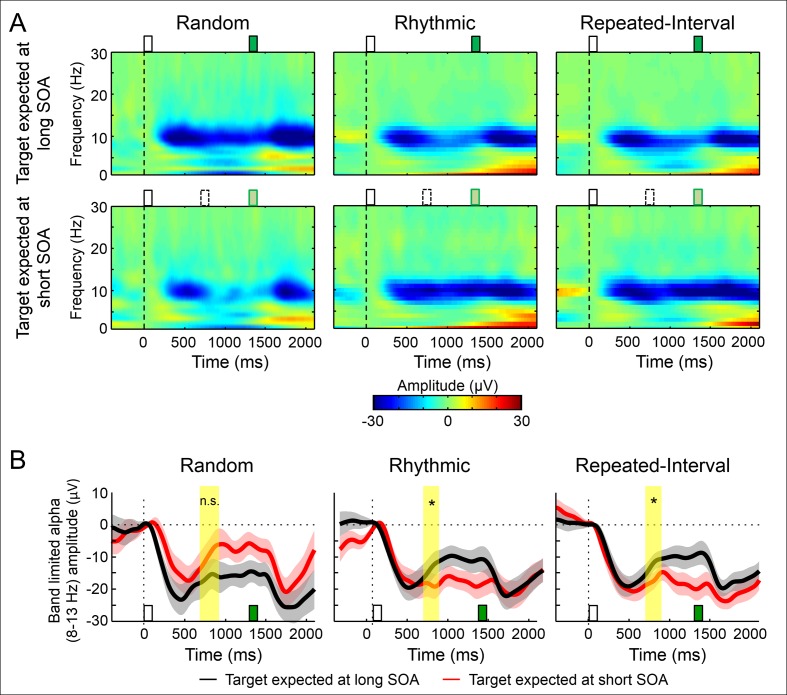
Similar amplitude modulation of occipital alpha-band activity by rhythm- and memory-based temporal predictions. (A) Time–frequency representations (group average, across six occipital electrodes) of the WS-target interval in the three experimental conditions for long SOA targets (green rectangle) when expecting the target in the short SOA, but it is omitted (bottom row, expected target in dashed) versus expecting it in the long SOA (top row). (B) Band-limited alpha amplitude (8–13 Hz). Error margins reflect the standard error for the difference between SOAs; Yellow shading—the predefined interval for analysis (*p < 0.05).

In our data, there were narrow-band amplitude modulations in the alpha range (Fig 4A). The most pronounced amplitude modulation was a sharp decrease of amplitude of the alpha band following the WS, which then recovered, a typical response to visual stimuli (Fig 4B). In the Rhythmic condition, when expecting a target at a short SOA, the recovery was blunted, resulting in lower amplitude in the short SOA window compared to when expecting a target at the long SOA (*t*_(18)_ = 1.80, *p* = 0.044, *d* = 0.41). This modulation was not explained by differences in the average stream SOA, as it was not observed in the nonpredictable Random condition, in which there was even a trend for higher alpha amplitude when expecting a target at the short SOA. However, similar amplitude modulation of alpha-band activity was observed in the Repeated-Interval *(t*_(18)_ = 1.82, *p* = 0.043, *d* = 0.42) condition, and an ANOVA conducted across the predictive conditions revealed no interaction between SOA and condition (*F*_(1,18)_ = 0.14, *p* = 0.71) but only a main effect of SOA (*F*_(1,18)_ = 5.23, *p* = 0.036). There was also no main effect of condition (*F*_(1,18)_ = 0.01, *p* = 0.92). Thus, the reduction of alpha-band amplitude at the time of an expected event did not depend on whether the prediction was rhythm or memory based.

#### Delta-band phase

Previous studies reported increased PC in the delta band at the time of rhythm stimuli, such that the optimal phase for performance aligns with rhythmic stimuli times [3,6,7,21,22]. This effect was interpreted as reflecting entrainment of intrinsic oscillations to the rhythm. Under this premise, PC should be stronger in the Rhythmic relative to the Random and Repeated-Interval conditions, since, in the latter two, the stream is less periodic and hence less able to entrain oscillatory activity. To test this, we extracted the phase of delta-band activity (0.5–3 Hz) from occipital electrodes at target time in the three experimental conditions and, as additional control, at the time of the WS in the Rhythmic condition. The Random condition target was used as a control in which there was preparation for a target but no specific temporal prediction. The WS in the Rhythmic condition was used as a control in which a task-relevant event (the WS) is temporally predictable, but there is no preparation to respond to it.

**Fig 5:**
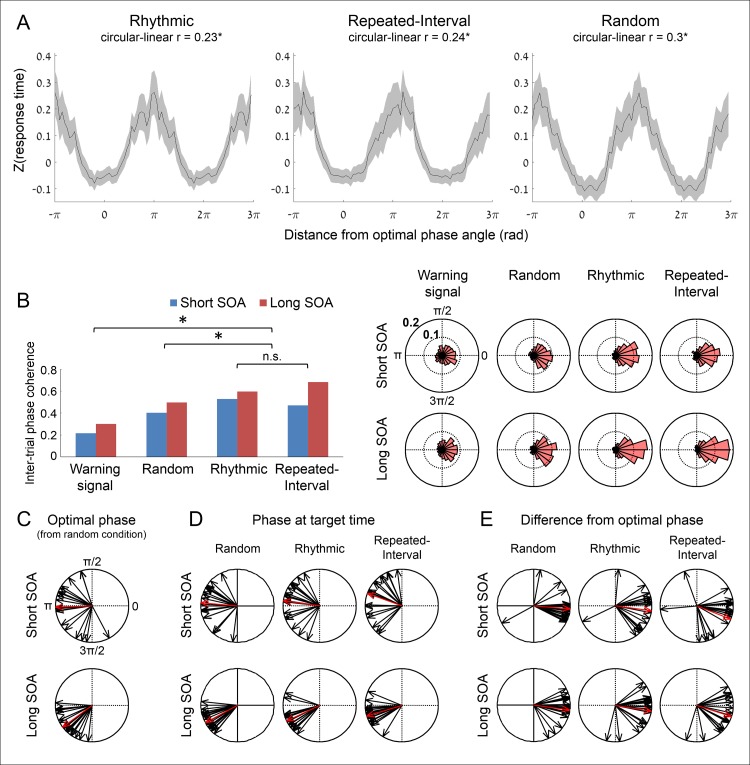
Similar phase modulation of occipital delta-band activity (0.5–3 Hz) by rhythm- and memory-based temporal predictions. (A) Association between delta phase and performance in the three experimental conditions. Standardized RT as a function of distance from optimal delta phase at target time, pooled across all trials and smoothed with a moving π/2 wide window (two identical waveforms concatenated for visualization). To accumulate across subjects, we standardized RTs within subject and normalized phases to have an optimal phase of zero. *p < 0.05. (B) Inter-trial phase coherence (ITPC) values and polar histograms of delta phase in the two predictive conditions at target time, and in the two control conditions, at target time in the Random condition and at WS time in the Rhythmic condition. *p < 0.05. (C) Optimal delta phase angles in short and long target SOAs for each participant (black) and across participants (red). (D) Delta phase angles at target time in the three experimental conditions in short and long target SOAs, averaged across trials for single participants (black) and across participants (red). (E) Differences between average delta phase angles at target time and the optimal angles for single participants (black) and group (red). A difference of zero implies that the phase at target time was identical to the optimal phase.

We first examined whether there is an optimal delta phase for performance, that is, a correlation between delta phase at target time and RTs. Such correlation is essential for entrainment accounts because if performance is not associated with the phase of oscillations, there is no benefit from aligning the oscillation phase with expected times. Plotting RTs as a function of delta phase across subjects revealed a circular–linear association in all conditions (sinusoidal instead of straight line, Fig 5A, compare with the much weaker correlation between RT and delta phase at the time of the WS in the Rhythmic condition, S1 Fig and S1 Text). Finding the phase–RT correlation in the Random condition is essential, as it indicates that this correlation is not merely driven by phase correction in the predictive conditions (Fig 5A, right panel). To test this correlation across all subjects while allowing a different optimal angle for each subject, we used a linear mixed-effects model that predicted single trial RTs from delta phase (represented as its sine and cosine) and target SOA in the Random condition (see S1 Text). This analysis revealed a significant fixed effect for delta phase ($\chi_{\left(2\right)}^{2}=\text{7}.\text{82}$, *p* = 0.02) but also a significant effect for the random slopes of delta phase ($\chi_{\left(5\right)}^{2}=\text{15}.\text{16}$, *p* = 0.01), suggesting that delta phase was associated to RT with a different optimal phase for each subject. Optimal phase did not differ between short and long SOAs ($\chi_{\left(1\right)}^{2}=0.0\text{6}$, *p* = 0.81). Thus, the phase of delta activity at target time was associated with RT, in accordance with the idea that this activity reflects fluctuations in efficiency of neural processing. This also indicated that performance is optimal in selected portions of the delta cycle.

Next, we tested whether rhythm-based predictions lead to increased PC at the time of the target relative to memory-based predictions or with no temporal information. If delta PC reflects entrainment of low-frequency oscillations, the inter-trial phase coherence (ITPC) should be stronger in the Rhythmic condition compared to the other conditions. For this purpose, we calculated the ITPC at target time (Fig 5B), and compared it between conditions using an omnibus permutation-based ANOVA that does not require the parametric assumptions of conventional ANOVA (see S1 Text). This analysis revealed a main effect of condition (*p* < 0.01). Planned contrasts revealed that the ITPC was increased in the Rhythmic condition compared to the Random condition (*p* < 0.01) and compared to the WS in the Rhythmic condition (*p* < 0.01). However, the ITPC was also increased in the Repeated-Interval condition compared to the Random condition (*p* < 0.01) and compared to the WS in the Rhythmic condition (*p* < 0.01). Crucially, the ITPC did not differ between the Rhythmic and Repeated-Interval conditions (*p* > 0.5, CI for the ITPC difference: −0.061 to 0.035). The ANOVA also revealed stronger ITPC for targets appearing at long compared to short SOA (*p* < 0.01) and a significant interaction between target SOA and condition (*p* < 0.05). Permutation-based *t* tests revealed that for short target SOA, there was a nonsignificant trend for the ITPC to be stronger for the Rhythmic compared to the Repeated-Interval condition (*p* = 0.1), while for long target SOA, the ITPC was in fact weaker for the Rhythmic compared to the Repeated-Interval condition (*p* < 0.01). Thus, enhancement of delta PC was not unique to rhythm-based temporal predictions.

Finally, we examined whether the delta-band PC is around the optimal phase for performance. It could be the case that memory-based predictions lead to increase in delta PC, but not towards the optimal angle, which would suggest that this PC reflects a different process than the one that takes place when predictions are based on rhythms. First, we estimated the optimal phase for performance of each subject by averaging the phases of the fastest 33% of the trials in the Random condition (Fig 5C). Next, we calculated for each subject in each condition the average phase at target time (Fig 5D). If the phase at target time was shifted to the optimal angle, the difference between each subject’s optimal phase and phase at target time should have an expected value of zero. Fig 5E presents these differences for all subjects in each condition, demonstrating a distribution around zero. To quantify this, we used a circular statistical test, termed *V* test, which tests whether phases are unimodally distributed around an a priori specified angle (in contrast to uniformly distributed). When using zero as the a priori angle, this test was significant in both short and long SOAs in the Rhythmic (short *V* = 14.04, long *V* = 15.54, all *p* < 0.01) and similarly in the Repeated-Interval (short *V* = 11.84, long *V* = 15.03, all *p* < 0.01) conditions. Thus, the distribution of the circular distances between the phase angle at target time and the optimal phase for each subject was significantly concentrated around zero, indicating alignment with the optimal phase in both predictive conditions (Fig 5C). In summary, the delta PC and alignment with the optimal phase observed in the Rhythmic condition were observed at least to a similar extent in the Repeated-Interval condition, implying that these effects were not unique to rhythms.

#### Target-evoked P3

**Fig 6:**
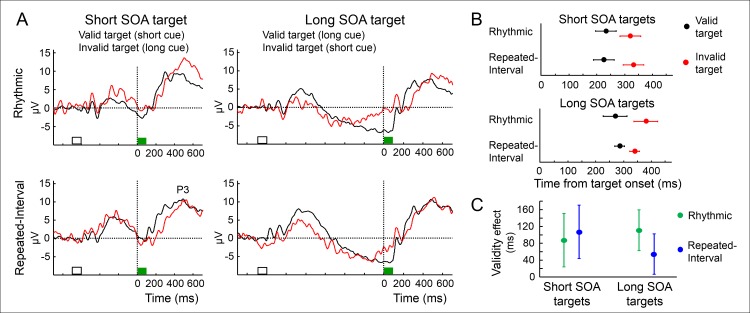
Similar modulation of P3 latency by rhythm- and memory-based temporal predictions. (A) Averaged waveforms in the two predictive conditions for validly (black) and invalidly (red) cued targets (green bar). Waveforms are averaged across a three-electrode parietal cluster and across participants locked to the target (green bar). Time zero is target onset. (B) Latency estimates of the P3, defined as the time point in which it reaches 50% of its amplitude. Error margins reflect one standard error of the difference between valid and invalid. (C) The effect of target validity on the P3 latency in each target SOA in the two predictive conditions. Error margins reflect one standard error of the difference between the predictive conditions in each SOA.

The P3 is a parietal-maximum ERP following target detection [41,42]. Its latency is shortened when target timing is expected compared to unexpected [26,34]. We examined whether this modulation differs between rhythm- and memory- based temporal predictions, separately for short and long SOA targets (due to pretarget CNV-related effects, Fig 6A). We used a jackknifing method (see S1 Text) to extract the latency in which the P3 reached 50% of its full amplitude and submitted them to a two-way ANOVA with factors Condition and Validity within each SOA. As rhythm-based predictions lead to stronger behavioral validity effect, it could have been expected that they will be reflected in a larger validity effect on the P3 latency. Overall, the P3 latency was shorter for valid compared to invalid targets both for short SOA targets (F_(1,18)_ = 24.57, p = 0.0001, Rhythmic: t_(18)_ = 2.35, p = 0.03, Repeated-Interval: t_(18)_ = 2.85, p = 0.01) and for long SOA targets (F_(1,18)_ = 13.38, p = 0.0017, Rhythmic: t_(18)_ = 2.57, p = 0.019, Repeated-Interval t_(18)_ = 2.97, p = 0.008, Fig 6B). Crucially, the validity effect did not differ between the Rhythmic and Repeated-Interval conditions (Validity X Condition interaction F_(1,18)_ = 0.93, p = 0.35 and F_(1,18)_ = 1.37, p = 0.26 for short and long SOA targets, Fig 6C). There was also no main effect of condition in either target SOAs (both F's < 1, both p > 0.3). Thus, the modulation of the P3 latency by temporal predictions did not differ between rhythm-based and memory-based predictions.

### Oscillatory entrainment model

The Repeated-Interval condition was designed to hinder stable entrainment of neural oscillations relative to the Rhythmic condition (especially at the rhythm frequency) by presenting the first stimulus of each pair approximately on the opposite phase of the oscillation that is entrained by the previous pair yet to be equally informative of target timing based on interval-memorization. However, although the Repeated-Interval condition was much less periodic than the Rhythmic condition (as indicated by SOA variances, spectral amplitudes, and autocorrelation functions, Fig 1), the fact that it has a repeated interval introduces more power to some frequencies over others. For example, the even stimuli in the Repeated-Interval condition create a meta-stream at a sub-delta band that is somewhat periodic (standard deviation of 5%–7% of the mean interval). Notably, entraining to such meta-stream would mean “skipping” the WS (which was an odd stimulus) and relying on a noisy source for prediction rather than using the fully predictive interval. While in debriefing subjects reported using the interval and not applying such strategy, it could not be ruled out that some passive entrainment could have occurred.

Therefore, we implemented a computational oscillatory entrainment model and used it to estimate the ITPC values and angles that are predicted by entrainment to the stimulation streams. Especially, we examined whether entrainment to a slow periodicity could explain the similar levels of ITPC and similar mean angles observed in the EEG data between the Repeated-Interval and Rhythmic conditions. Following previous entrainment models [43,44], we used a multi-oscillator model which consisted of an array of oscillators with different frequencies in the delta range. The phase angle of each oscillator was modeled as a dynamical system that exhibits endogenous periodic activity at a specific frequency (the oscillator’s “natural frequency”) and follows phase-resetting towards the optimal phase for performance (defined as an angle of zero) with exposure to external transients. The stimulation stream was represented as a time series of impulse responses at the actual stimuli onset times. We used this model to obtain, for each trial, the expected angle that oscillators at each frequency would exhibit at target time (given an arbitrary, uniformly dispersed initial angle).

**Fig 7:**
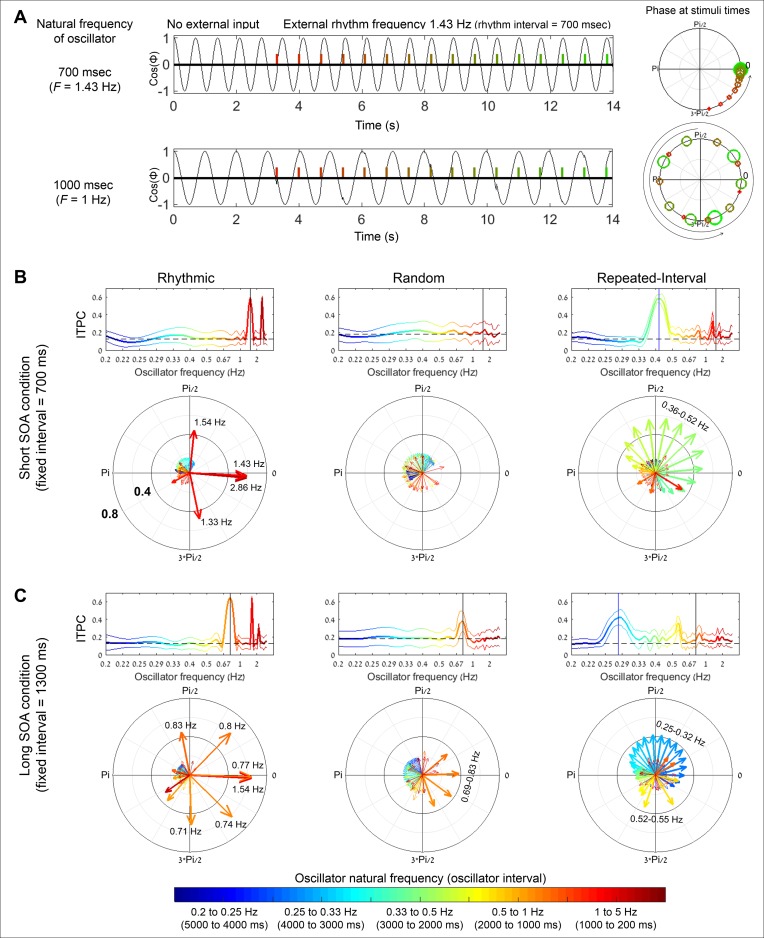
Predictions of an oscillatory entrainment model for the ITPC and angles in the experimental conditions. (A) Angle dynamics of two modelled individual oscillators (top row: with natural frequency of 1.43 Hz, and bottom row: with natural frequency of 1 Hz) entrained by the same rhythmic stimulus stream with frequency of 1.43Hz. Left: cos(*ϕ*) values as a function of time, first without an external entraining stream, then when exposed to the external entraining stream. Right: *ϕ* values at rhythmic stimuli times, color coded from red to green and from small to large with the progression of the stream along time. The stimuli entrain (phase-align) the oscillator with the matching natural frequency but not the one with a diverging frequency. (B) Modelled phase pattern of an array of oscillators with frequencies ranging from 0.2 to 5 Hz with exposure to the stimuli streams of the Rhythmic, Random, and Repeated-Interval conditions (left, middle, and right columns, respectively) with short SOA (700 ms, equivalent to 1.43 Hz). Different colors indicate the natural frequency of the oscillators. Top row: modelled ITPC values at target times (thick line) with 95% CIs (thin lines, Bonferroni corrected for multiple comparisons for the number of frequencies). Dashed horizontal line indicates the ITPC expected by chance, based on a surrogate distribution of ITPC with matching trial numbers. Black vertical line indicates the oscillator whose frequency matches the stream frequency (here 1.43 Hz). In the Repeated-Interval condition, a blue vertical line indicates the oscillator whose frequency matches the slow rhythm that is generated by every second stimulus (0.42 Hz). Bottom row: mean angles at target times across trials predicted by the model for oscillators with different natural frequencies. The length of the arrows represents the ITPC. The color of the arrows represents the natural frequency, corresponding to the colors in the top row. Frequencies with relatively high ITPC are annotated with numbers. (C) Same as B for the long SOA (1,300 ms, equivalent to 0.77 Hz). In the top row, black and blue vertical lines indicate oscillators with frequencies of 0.77 and 0.28 Hz, respectively.

The behavior (cosine of *ϕ* as a function of time) of two oscillators with a natural frequency of 1.43 Hz (top row) and 1 Hz (bottom row) in a short SOA (700 ms) Rhythmic condition trial is presented in Fig 7A. Prior to the introduction of an external stream (colored bars), both oscillators fluctuate with a stable phase. Presentation of external stimuli elicits phase correction, with the peak of the cycle (angle of zero) attracted towards alignment with the rhythmic stream. For the 1.43 Hz oscillator (which matches the rhythm SOA), this leads to phase stabilization relative to the stream. In contrast, for the 1 Hz oscillator, this leads to phase dispersion, as phase correction by one stimulus causes the subsequent stimulus to fall off-phase and elicit more phase correction. This behavior is also evident in the polar plots to the right, which illustrate the phase angles at stimuli time (the progression of the stream is coded by color change from red to green and size increase). Thus, this model successfully reproduces entrainment behavior.

We next obtained the ITPC and mean angle values expected in each condition by simulating this model for an array of oscillators with delta frequencies (0.2 Hz to 5 Hz, equivalent to cycle lengths of 5,000 to 200 ms in 50 ms steps) for all experimental trials, separately for each subject. Fig 7B presents the obtained ITPC values (top row) and mean angles (bottom row) at target times as a function of natural frequencies for the short SOA conditions. For the Rhythmic condition (left column, stimulation frequency 1.43 Hz), oscillatory entrainment leads to high ITPC for a 1.43 Hz oscillator (marked in a black line) with an angle of zero, as expected. Strong ITPC is also predicted for the harmonics of this frequency. For the Random condition (middle column), oscillatory entrainment results in relatively low ITPC for all oscillator frequencies. For the Repeated-Interval condition (right column), oscillatory entrainment predicts minimal ITPC at the 1.43 Hz frequency. Notably, some ITPC increase is predicted at a lower frequency of ~0.42 Hz, corresponding to the slow periodicity emerging between each second stimulus (interval of 2,400 ms). Crucially, however, the angle that is predicted at this frequency is not zero, thus the entrainment here would not lead to alignment of the optimal phase to stimuli times, in contrast to what was observed in the EEG results (similar levels of ITPC around the same optimal phase in the Rhythmic and Repeated-Interval conditions). In the long SOA condition (Fig 7C), the difference between conditions is even more pronounced, with a difference not only in mean angle but also in the level of ITPC (stronger in the Rhythmic condition). Thus, the low-frequency oscillation in the Repeated-Interval condition cannot account for the similar ITPC with alignment to a phase of zero observed in the EEG data for both conditions.

## Discussion

The current study compared predictions formed in a rhythmic context to predictions formed by interval memorization in streams that are less rhythmic, in order to dissociate these two prediction modes and put the putative manifestations of oscillatory entrainment to a critical test. We found that PC and alignment of delta-band EEG activity did not differ between the Rhythmic and Repeated-Interval conditions. Thus, these measures are not good indicators of entrainment mechanisms and could reflect memory-based predictions. This was also the case for behavioral facilitation for expected targets, modulations of alpha amplitude and post-target P3 latency. However, we also found that rhythm-based predictions resulted in slower responses to unexpected targets than memory-based predictions and in immediate resolution of the CNV when a temporal prediction was unexpectedly violated. These possibly reflect a resonance mechanism compatible with entrainment. Our findings thus suggest that temporal predictions in rhythmic contexts is a combination of memory-based and entrainment-like mechanisms, affecting different manifestations of performance and electrophysiology. Crucially, they imply that resonance after stream termination, and not PC during the stream, is the indicator of rhythm entrainment.

### PC and alignment of slow EEG oscillations is not a unique signature of rhythm entrainment

Delta-band activity at rhythmic stimuli times demonstrates enhanced PC around the optimal angle for performance relative, e.g., to aperiodic nonpredictive streams [3,6,7,21,22]. This phenomenon, also replicated in our data, is commonly interpreted as reflecting entrainment of intrinsic low-frequency neural oscillators to the external rhythm [10,11,23,44]. However, PC was also increased in our aperiodic yet predictable condition (Repeated-Interval condition) relative to an aperiodic nonpredictable condition (Random condition). In fact, the magnitude of PC in the Repeated-Interval condition did not differ from the Rhythmic condition, although the former was much less rhythmic. Thus, PC cannot be taken as conclusive evidence for oscillatory entrainment to rhythm without controlling for other sources of PC.

If similar delta PC is observed in streams that clearly promote oscillatory entrainment and streams that are more detrimental to entrainment, what could be the source of PC? One plausible alternative is the temporal predictability of the streams, which is inherent to rhythms [33,34] and was matched in our study between the Rhythmic and Repeated-Interval conditions. Neurophysiologically, temporal predictability is known to drive buildup of neuronal activity to peak at the expected time point and then resolve [30,31,38], a phenomenon that is often referred to as climbing neuronal activity (CNA) [45,46]. This pattern produces a waveform with a wavelength matching the expected interval, which, as the rhythm interval is in the delta range, seems like an apparent delta cycle (see also [21], for acknowledging this possibility). Since accurate temporal prediction means that the peak repeatedly coincides with the stimulus, apparent PC is observed around stimulus time. Furthermore, PC is observed around the optimal phase because the CNA peak coincides with the time in which temporally driven preparation is maximal, making it seem as an optimal phase. It was recently argued that a CNA cannot account for entrainment effects, as PC did not increase in conditions in which the CNV amplitude was expected to increase [47]. However, phase consistency could in fact be independent of amplitude. Regardless, the present findings directly show that rhythmicity is not needed for apparent delta PC and that it could be well explained by CNA. Our study relies on scalp EEG but even with finer spatial resolution methods (e.g., electrocorticography), the critical comparison we present here is needed to disentangle entrainment from repeating climbing activity.

The source of the CNA recorded in occipital channels is yet to be determined. Several lines of evidence point to a key role of the motor system in timing and in the generation of CNA, intracranially [48–50] or on the scalp as the CNV [38]. Possibly, the observed occipital CNA reflects volume-conducted CNV originating in premotor regions. Notably, this caveat applies to all EEG studies of visual and especially auditory rhythms, as auditory responses and the CNV are measured in the same sites. However, the motor system has also been associated with predictions even when these are nonmotor [51] and was suggested to be involved in purely sensory temporal predictions [52–54]. Thus, another possibility is that the occipital CNA may result from top-down signals from the motor system that prepare the relevant sensory cortex at the right time [55,56]. A final possibility is that the occipital CNA originates from nonmotor duration representations, for example in parietal regions [57], or even localized timing mechanisms in the sensory cortex itself [58].

Further support for PC not being due to continuous rhythm entrainment comes from the finding that PC was low at the time of the WS, despite the WS being embedded in a rhythmic stream, anticipated, and task-relevant (as it signaled an upcoming target, see also Fig 2A and 2B for absence of CNV or any oscillatory trace prior to the WS). We speculate that in paradigms in which there is no WS and all rhythmic stimuli are possible targets (e.g., [6,22]), CNA is elicited after each stimulus, resulting in apparent continuous oscillation.

Importantly, the current findings do not refute the existence of low-frequency oscillations, their role in sensory selection, or the possibility that they become entrained to external rhythms. However, we argue that phase modulations, whether increased concentration or angle reversal, of filtered neural signal during the presentation of the stimulus stream cannot be taken as evidence for the existence or entrainment of such oscillations. This argument is agnostic to the question of whether the climbing activity (and in particular the CNV) is a result of phase-resetting of ongoing low-frequency oscillations by the WS [11,21] (see also [59]). However, in the Repeated-Interval condition such phase-resetting should be selective for the oscillator with the wavelength of the expected interval, based on memory, rather than on bottom-up entrainment.

Interval timing loses its efficiency for intervals outside the range of 200–2,000 ms (i.e., the delta range), [60]. Thus, it is less likely that the effects of rhythmic stimulation at faster rates, especially alpha in visual perception [18], can be explained by interval timing. Given the prominence of alpha rhythms in visual cortex and its hypothesized relation to the timing of neural activation [61], it is possible that effects of alpha-rate stimulation are indeed mediated by entrainment of alpha oscillations, aligning stimuli with a preferred phase of the alpha cycle [14,15]. Future studies should examine the mechanism of alpha entrainment using appropriate control for temporal predictability as used here.

### Rhythmic prediction mechanisms obligate resource withdrawal at unexpected times

Previous studies separately found behavioral validity effects for rhythms and for memory-based predictions [16,25]. Rhythm processing is supported by the motor system, especially the supplementary motor area [62,63]. Given the manual nature of our task, it was plausible to expect an advantage of rhythm-based over memory-based predictions. Indeed, rhythms exerted a stronger expectation, but this was expressed in larger cost of prediction violation, rather than in stronger facilitation for expected targets. This suggests a dissociation between the processes involved in increasing preparation at expected times and relative inhibition at unexpected times instead of a tradeoff of the same resource between the expected and unexpected times. The pattern of CNV resolution when the anticipated event was unexpectedly omitted provides an apparent neural correlate for the extra cost of unexpected events (Fig 3). When a short SOA target was expected but did not appear, the CNV resolved immediately towards baseline with rhythm-based predictions (compare with [9]) but only after a delay for memory-based predictions (compare with [37]).

The interpretation of this pattern depends on the process reflected by the CNV, an issue that is still under debate [64]. Under the model that the CNV reflect the accumulation of pulses of a timer mechanism [37,65], the CNV resolution reflects "emptying" of the accumulator once the memorized interval has elapsed and a decision has been made. In this context, the delayed resolution of the CNV in the Repeated-Interval condition could result from reduced temporal accuracy of memory-based predictions, which rely on multiple factors like working memory, arousal, and attention [28,66]. This would lead to more jitter in the peak latency of the accumulation at the end of the expected interval and, as a byproduct, to “leakage” of preparation to times other than the expected time.

However, the timer model of CNV is challenged by recent arguments that the CNV resolution is modulated according to optimal decision strategies, which suggests that the CNV is more likely to reflect active preparation than mere passive timing [64,67,68]. Thus, the difference between our predictive conditions in the CNV resolution, as well as the RT results, imply that memory-based predictions are more flexible, allowing dynamic shifting of resources to unexpected times when a prediction is violated. Rhythmic predictions on the other hand are obligating, with inability to maintain a high level of preparation in between rhythmic stimuli. This obligatory nature of the rhythmic condition is in line with our previous finding, showing that rhythms affect the CNV even when they are not predictive and not intentionally used to form predictions [34].

### Modulations of alpha-band oscillations and posttarget P3 reflect general temporal predictions regardless of source

Earlier P3 latency for temporally expected compared to unexpected events was previously observed in both rhythmic [34,36] and memory-based paradigms [26], although the two modes were not directly compared until now. Since the P3 has been related to stimulus evaluation and response selection [41,42], the earlier P3 for temporally expected stimuli was interpreted as reflecting sharpening of such processes due to preparation. Our results, showing similar effects of rhythmic and memory-based predictions, imply that this sharpening results from intentional usage of available temporal information, independent of its source.

Previous studies found amplitude reduction of occipital alpha-band oscillations before or at the expected time of a target embedded in a rhythmic stream [9,34,35]. Our findings suggest that this phenomenon (presumably indexing increased perceptual excitability) is a signature of temporally focused preparation in general. Anticipatory reduction of occipital alpha-band amplitude was also observed in paradigms in which a nontemporal feature was anticipated, such as in reduction of alpha power in electrodes contralateral to an expected target location [69,70]. While spatial information allows biasing the cortical excitability to match the expected location (e.g., across hemifields), temporal information allows the increase of cortical excitability to focus at the expected time, possibly sparing resources. This may underlie synergistic effects of temporal and spatial predictions [8,71].

We previously showed that P3 and alpha modulations occurred only when intentionally using rhythms to form predictions but not incidentally (in contrast to CNV effects that were also observed with passive exposure to nonpredictive rhythms, [34]). Taken together, our current and previous results imply that the alpha-band and P3 effects reflect general mechanisms of intentional (rather than incidental) execution of temporal predictions, regardless of source.

### Three mechanistically distinct modes of dynamic resource allocation

Models of dynamic resource allocation argue for a dichotomy between temporally selective anticipation in rhythmic streams, mediated by oscillatory entrainment (“rhythmic” mode), and anticipation in nonrhythmic streams, which requires sustained vigilance (“vigilance” mode) [11]. These models leave out the well-documented memory-based resource allocation in time, which was shown to occur without rhythmic context [1,26]. Moreover, it was not clear whether rhythm- and memory-based temporal predictions are distinct at all, as interval-timing neural signatures were explained by oscillatory phase resetting [11], and, in contrast, seeming oscillations could be explained by repeated interval-timing [32] or even repeated evoked responses [72]. Similarly, imaging studies revealed that both rhythm- and memory-based temporal predictions activate similar regions (premotor and inferior parietal cortices, for a review see [73]).

Directly comparing conditions in which timing is predictable, based on memory, rhythm, and not at all, allows us to extend the dichotomy into a trichotomy: availability of temporal information, regardless of its source, allows the brain to shift from the “continuous” mode into a “temporally predictive” mode. This is characterized by pretarget CNA (such as the CNV), anticipatory alpha reduction, and posttarget facilitation of processing of temporally expected events, reflected by earlier P3 latency and shortened RTs. When anticipation is based on rhythms, the brain can further switch into a “rhythmic” mode, characterized by similar manifestations but also by immediate cessation of facilitation and possibly inhibition once the expected time interval has elapsed, manifested by CNV immediate resolution and larger behavioral costs for unexpected events. Methodologically, studies that aim to infer rhythm-specific mechanisms should use a less rhythmic predictive control similar to our Repeated-Interval condition, which would allow attributing the effects of rhythms to rhythmicity and not to general temporal prediction.

### Rhythm-based predictions uniquely associated with resonance-like pattern

A central remaining question is whether the unique effects of rhythms are mediated by oscillatory entrainment. On one hand, this possibility is weakened by our finding that PC of low-frequency oscillations, which were usually conceived as a signature of rhythm-specific entrainment, was observed to the same extent in less-rhythmic streams. On the other hand, oscillations are characterized by resonance, that is, extension of several oscillatory cycles even when external phase-resetting input is terminated. Findings fluctuations of several cycles in behavioral and physiological markers after stimulus termination was often interpreted as a direct evidence for the alignment of a self-sustaining oscillatory process to the external rhythm [74–76]. In our data, the conspicuous and immediate return of the CNV to baseline after an omission, in the absence of any external event to phase-reset it, represents the expected pattern of a resonance mechanism, consistent with entrainment. It could also explain the increased cost of presenting targets outside the expected time window.

More than the ability to recruit resources, what seems to be unique to the rhythmic mode (and not to the anticipatory mode in general) is the tendency to “turn off” anticipation periodically, consistent with the idea of energy efficiency [11]. While this mode is indeed efficient when the input maintains its predictability, the current findings also emphasize the inflexibility and resultant cost of this mode when events are temporally surprising. This may be an unavoidable consequence of the oscillatory mode.

## Materials and methods

### Ethics statement

This study and all of its procedures, including subject recruitment methods, instructions, experimental task, apparatus, debriefing, and reimbursement, were approved by the local Ethics Committee of the Hebrew University of Jerusalem, Israel. All participants provided written informed consent according to the local Ethics Committee and the Declaration of Helsinki.

### Participants

Twenty-one students (11 females, mean age = 25, 19 right handed) were tested in return for course credit or payment. Participants reported no history of neurological or psychiatric disease, normal or corrected-to-normal vision, and no professional musical training or playing an instrument.

### Stimuli and design

Stimuli were colored disks (diameter 1.2°) presented for 100 ms. Each trial began with a preparatory sequence of stimuli, followed by a white WS and a green target to which participants had to respond with a speeded button press. A fixation point (black "+", 0.6° X 0.6°) preceded each trial for 500 ms. There were three conditions presented in different blocks (Fig 1A). In “Rhythmic” blocks, the sequence consisted of four, five, or six (uniform probability) black stimuli presented rhythmically with an SOA of 700 ms (1.43 Hz, short cue) or 1,300 ms (0.77 Hz, long cue). The WS appeared in-phase with the rhythm. The WS-Target SOA was identical to the rhythm SOA in valid trials or to the other SOA in invalid trials. In the “Repeated-Interval” condition, the sequence consisted of four, five, or six (uniform probability) black-then-red pairs of stimuli. The within-pair SOA was 700 and 1,300 ms in short and long cue trials, respectively, but the SOA between pairs was jittered (short trials: 1,500–1,900 ms, 50 ms steps; long trials: 1,900–2,700 ms, 100 ms steps, uniform distribution). We assumed that this aperiodic sequence structure would reduce the ability of endogenous delta oscillators to maintain a stable phase relation to the stream, as the first stimulus of each pair falls approximately on the opposite phase of the oscillation that is entrained by the previous pair. The last pair of the sequence consisted of a white WS followed by a green target. The SOA within this pair was the same as in the preceding pairs in valid trials and had the other SOA in invalid trials. In both Rhythmic and Repeated-Interval conditions, the sequences were predictive of target timing, as 75% of the trials within a block were valid and 12.5% invalid. In the remaining 12.5% of the trials, in each condition no target appeared following the WS, in order to diminish expectations based on conditional probability that the stimulus will occur given that it has not occurred yet [25]. In these “catch” trials, a blank screen was displayed after the WS for 1,500 ms. Finally, in the “Random” condition, the sequence consisted of four, five, or six (uniform probability) stimuli whose SOAs were jittered around the average of 700 or 1,300 ms (−200 to +200 in 50 ms steps, uniform distribution). Target SOAs were also drawn from these short and long distributions, independently from the average sequence SOA [34]. While the sequence was not predictive, for analysis purposes, “valid” trials were defined as those in which cue and target SOAs were drawn from the same distributions (e.g., short cue trials followed by short SOA targets, 43.75% of the trials) and “invalid” trials as those in which cue and target SOAs were drawn from different distributions (e.g., short cue trials followed by long SOA targets, 43.75% of the trials). The other 12.5% of the trials were catch trials.

### Procedure

The experiment was conducted in a sound attenuated chamber (Eckel C-26, United Kingdom). Stimuli were presented at the center of a 17-in CRT screen (ViewSonic G75f, with 100 Hz refresh rate) on gray background (viewing distance = 90 cm). Stimulus presentation and response acquisition were handled using Psychophysics toolbox [77,78] for MATLAB (version 7.5.0, Mathworks, Natick, Massachusetts). Trials were presented in blocks of 32 trials. The experiment started with two Random blocks. Next, Repeated-Interval and Rhythmic blocks alternated (four each). Which condition started was counter-balanced across participants. Participants were encouraged to use the predictive information if available. Eight practice trials were performed on first encounter with each condition and at least two trials for ensuing blocks. Short breaks were given between blocks. At the end of the experiment, participants were debriefed, verifying that they had used the predictive cues to create predictions. Prior to the entire experiment, all participants conducted three blocks of a condition from our previous study (“Non-Informative” condition) [34] for replication purposes. This condition and its results will not be presented in the main text (see S1 Text, and [34]).

### Behavioral analysis

For each participant, trials were discarded if the RT was larger than three standard deviations from the mean RT, separately for each condition and validity of the cue, or if the RT was shorter than 50 ms. RTs were subjected to a repeated-measures ANOVA with factors condition (Random/Rhythmic/Repeated-Interval), cue validity (valid/invalid), and target SOA (short/long). Where the sphericity assumption was violated, we report Greenhouse–Geisser corrected *p*-value and uncorrected degrees of freedom. Due to the small number of trials at the exact in-phase SOA in the Random condition, trials with SOA of up to 100 ms around the short and long target SOAs were used as representing these SOAs. The range of this interval (50–150 ms) did not affect the pattern of results. To examine the benefits and costs of having temporal predictions, we separately analyzed valid and invalid trials, conducting planned one-tailed contrasts to compare each predictive condition to the random condition, and two-tailed contrasts to compare the predictive conditions.

### EEG recording and preprocessing

EEG was recorded continuously from 64 preamplified Ag/AgCl electrodes, using an Active 2 system (BioSemi, The Netherlands) mounted on an elastic cap according to the extended 10–20 system. Additional electrodes were placed on the outer canthi of the right and left eyes and above and below the center of the right eye to track electro-oculographic activity, on the left and right mastoids, and near tip of the nose. The EEG signal was sampled at a rate of 1,024 Hz (24 bits/channel) with an online anti-aliasing 204 Hz low-pass filter. EEG preprocessing was conducted using BrainVision Analyzer 2.0 (Brain Products, Germany) using the following pipeline: referencing to the nose electrode; high-pass filtering using a zero-shift Butterworth filter with a cutoff of 0.1 Hz (24 dB/octave); correction of ocular artifacts using independent component analysis (ICA) [79] based on typical scalp topography and time course; elimination of epochs which contained other artifacts, defined as absolute activity larger than 100 μV, or a change of more than 100 μV in a 200-ms interval. The data of two participants were rejected from EEG analyses due to excessive artifacts.

### ERP analysis

ERPs were analyzed separately for the preparatory period, i.e., during the presentation of the stimulus sequence, and the posttarget period. For all ERP analyses, the data was re-referenced to the average of the left and right mastoids. In the preparatory period, the analysis was conducted in two predefined electrode clusters: a central cluster (FC1, FCz, FC2, C1, Cz, C2, CP1, CPz, CP2), to test for modulations of the CNV potential, and an occipital cluster (PO3/PO4, PO7/PO8, O1/O2), to test oscillatory activity in visual circuits. To visualize ongoing activity in these two clusters, segments extending from 5,600 or 3,200 ms before the target (for long and short SOA trials, respectively) to 1,000 ms after the target were averaged within condition across trials and subjects.

Two a priori hypotheses were tested regarding the CNV in the central electrode cluster. First, we examined whether the modulation of the CNV buildup by the expected SOA (i.e., faster buildup as the expected SOA is shorter, [9,26]) differs between conditions. For this, segments extending from 200 ms before to 1,300 ms after the WS were averaged within each condition for valid trials, separately for short and long cue SOA (in the Random condition according to the average of the sequence), with the period of 200 ms before the WS used as baseline. In the Random condition, we only used trials in which the target did not incidentally appear in the first 700 or 1,300 ms after the WS, for short and long SOA trials, respectively. Within each condition, the average CNV amplitude in a window of 400 to 700 ms after the WS was compared between expecting the target in short and long SOAs. This time window was chosen to provide maximal differentiation, as it was at the end of the short interval but in the middle of the long interval. Planned one-tailed contrasts were used to examine the hypothesis that the CNV will be more negative when expecting the target at a short SOA than long SOA.

Second, we compared the resolution of the CNV (i.e., return to baseline, [9,37,40]) between the different conditions in trials where an expected short SOA target was omitted (i.e., short-cue, invalid trials). In this situation, the timed interval ends, but no target appears to end the anticipation. For this, segments extending from 200 ms before to 1,400 ms after the WS were averaged separately for the Rhythmic and Repeated-Interval conditions, with the period of 200 ms before the WS used as baseline. To quantify the CNV resolution, we fitted the amplitude of the CNV waveform (averaged across participants) in each condition with a five-parameter continuous model, which allowed the CNV to buildup, remain at the peak amplitude for a flexible interval, and only then resolve (Fig 3C):

$$Amp\left(t\right)=\{\begin{array}{cc} 0 & 0<t\leq T_{0}\\ S_{1}*\left(t-T_{0}\right) & T_{0}<t\leq T_{1}\\ peak\_amp & T_{1}<t\leq T_{2}\\ peak\_amp+S_{2}*\left(t-T_{2}\right) & T_{2}<t\leq 1400 \end{array}$$

$$peak\_amp=\;S_{1}*\left(T_{1}-T_{0}\right)$$

The free parameters of the model were the buildup initiation time (T_0_), buildup slope (S_1_), buildup termination time (T_1_), resolution initiation time (T_2_), and resolution slope (S_2_). The possibility for a variable delay between T_1_ and T_2_ allowed accurate estimation that would not be distorted by predefined choice of intervals and also reflected a cognitively plausible strategy of maintaining high preparation after target omission, in case the target appears at the long SOA. Each parameter was compared between the Rhythmic and Repeated-Interval conditions using paired *t* tests using the jackknifing procedure (see S1 Text). To verify that the five-parameter model does not overfit the data, this “resolution after delay” model was compared to a more restrictive four-parameter model, “immediate resolution," in which the transition between buildup termination and resolution initiation was immediate (T_1_ = T_2_, see Fig 3D). Model fitting was conducted using the “fminsearch” function in MATLAB (Nelder–Mead simplex optimization) with several initial values. Model selection was based on calculation of the Bayesian Information Criterion (BIC, [80]). For each condition, the model with the lowest BIC value was chosen (ΔBIC > 6 is considered strong evidence).

ERP analysis of posttarget activity focused on the P3 potential, which was analyzed at a cluster of midline-parietal electrodes (Pz, P1, P2). We examined whether the modulation of the P3 latency by the validity of temporal prediction (shorter latency when the target appears at the expected SOA, [8,26]) differs between conditions. For this, segments extending from 200 ms before to 700 ms after the target were averaged in each predictive condition, separately for trials with short or long target SOA and for valid or invalid cues. As the pretarget CNV effect was expected to result in differences in the pretarget baseline in short versus long target SOAs, we used a period of 200 ms before the WS as baseline and analyzed each target SOA separately. The time window for P3 analysis was 200–550 ms after target onset, based on unbiased visual inspection of average activity across all subjects and conditions. The P3 latency was estimated by averaging the latencies in which the amplitude reached 30%, 50%, and 70% of the peak amplitude, allowing a stable estimation of the half-maximum point [81]. Reliable estimation of these latencies was achieved using a jackknifing procedure. Within each condition and target SOA, the amplitudes and latencies of the P3 were each subjected to a repeated-measures ANOVA with factors condition (Rhythmic/Repeated Interval) and target validity (valid/invalid), and planned one-tailed contrasts were used to examine whether the P3 latency is shorter when the target SOA was expected compared to unexpected.

### Alpha-band amplitude analysis

Following previous findings [9,34,35], we analyzed amplitude modulations of alpha-band activity (8–13 Hz) in occipital electrodes (PO3/PO4, PO7/PO8, O1/O2) by temporal predictions. We compared the amplitude of alpha activity at the time of the short SOA target when expecting the target at the short versus long SOA. In both short and long SOA trials, we only included trials in which the target actually appeared at the long SOA, avoiding contamination by target-evoked activity. Thus, the only difference between the trials was the predicted target time. We asked whether an amplitude reduction at the time of a temporally expected target would differ between the different sequences. The data were segmented from 1,400 ms before the WS to 1,800 ms after the target. Instantaneous amplitudes were extracted using a complex Morlet wavelet transform (1–30 Hz, 1 Hz steps, ratio between the central frequency and the standard deviation of the Gaussian-shaped wavelet in the frequency domain = 8). The first and last 1,000 ms of each segment absorbed edge artifacts and were discarded from analysis. The resulting time–frequency representations were averaged across electrodes within each condition. Baseline correction was performed using a range of 0–100 ms after the WS, as the occurrence of the WS is the only time point that is identical both in function and in perceptual input between the short and long SOA conditions (pre-WS intervals were contaminated with residual activity from previous stimuli, see [34]). Average alpha-band amplitude (across 8–13 Hz and 700–900 ms after the WS) was subjected to a repeated-measures condition (Rhythmic/Repeated-Interval) X expected SOA (short/long) ANOVA. Within each condition, planned one-tailed contrasts examined whether the alpha-band amplitude is reduced when the target was expected at the short SOA compared to the long SOA.

### Delta-band phase analysis

We analyzed the effects of temporal predictions on the phase of delta-band activity in occipital electrodes in which these effects were expected to be maximal (PO3/PO4, PO7/PO8, O1/O2). We examined whether increased PC and alignment to the optimal phase angle at target time [3,6,7,21,22] occur in the rhythmic and Repeated-Interval conditions. Instantaneous phase of delta-band activity was extracted by band-pass filtering the unsegmented data in a frequency band of 0.5–3 Hz (zero-shift Butterworth filter, 24 dB/octave) and applying the Hilbert transform. This was performed separately for each occipital electrode and then averaged (considering the circularity of phase) across electrodes. The delta-phase signal was segmented into epochs surrounding valid targets (−700 to 500 ms for short SOA targets and −1,300 to 500 ms for long SOA targets), separately for the Rhythmic, Repeated-Interval, and Random conditions and, for comparison, also surrounding the WS in the Rhythmic condition. Analyses of circular variables were performed using the CircStat toolbox for MATLAB [82].

To examine whether certain phase angles were optimal for performance we tested the circular–linear association between delta phase at target time and RTs in the random condition (as in the predictive conditions delta-phase was expected to have small variance due to PC). We used a linear mixed-effect regression model to estimate this association at the group level, allowing each subject to have a different optimal value (see S1 Text). Delta phase was linearized by taking its sine and cosine and using them as fixed effects along with target SOA. The intercepts of participants, as well as by-participant random slopes for sine and cosine of the phase, were inserted as random effects.

Second, we examined delta PC at stimulus time (target/WS) by calculating the ITPC [83]. Across participants, these ITPC values were compared between conditions using an omnibus repeated-measures ANOVA with factors condition (Rhythmic/Repeated-Interval/Random/WS-Control) and SOA (short/long), followed by subsidiary ANOVAs for comparing specific condition pairs. Specifically, we examined the occurrence of ITPC in the predictive conditions by comparing them to the controls, and also compared the Rhythmic and the Repeated-Interval conditions. Due to the nonnormality of the ITPC data (bounded between zero and one), we used permutation-based ANOVAs (see S1 Text).

Finally, we examined whether delta phase at target time was aligned to the optimal phase angle, which was identified in each participant and each SOA by averaging the phases of the 33% fastest trials in the Random condition. For each predictive condition, we then calculated for each participant the circular distance between the circular-averaged phase angle at target time and his\her optimal phase. If the phase at target time was shifted to the optimal angle, the expectancy of these differences is zero, so they should be concentrated around zero across subjects. We tested this using a *V* test, which contrasts a null hypothesis that phases are uniformly distributed to an alternative hypothesis that phases are unimodally distributed with an a priori specified mean angle, which, in the current case, was set to be zero (see [7]). With the same approach, we also tested whether the phase angle at target time was identical between short and long SOA targets.

### Oscillatory entrainment model

To examine whether the observed EEG results are consistent with an oscillatory entrainment mechanism, we used a computational oscillatory entrainment model [43,44] to estimate the ITPC magnitude and mean angles that entrained neural oscillators are expected to show in the three experimental conditions. Following Large and colleagues [43,44], we used a model consisting of an array of neural oscillators with different characteristic frequencies in the delta range. Each neural oscillator was modeled using a dynamical system characterized by two state variables, phase angle (*ϕ*) and amplitude (*r*), and was coupled to an external stream (*s*) that was modeled as a sequence of transients at the times of stimuli onsets:

$$\begin{array}{l} \frac{d\phi }{dt}=2\pi F-s\left(t\right)*c*\sin\left(\phi \right)\\ \frac{dr}{dt}=0 \end{array}$$

In the absence of external stimulation (i.e., *s(t)* = 0), this system produces periodic dynamics, with the angle revolving steadily with time as a function of the *F*, the natural frequency of the oscillator. When a stimulus is presented (*s(t)* = 1), an additional term corrects the angle towards zero, which is assumed to be the angle of the external stream. The strength of correction is determined by a coupling parameter *c*, which was adjusted such that the modeled ITPC levels in the Rhythmic condition would be similar to those observed in the actual data. Given a set of initial conditions, this system can be numerically integrated to examine its convergence to a limit cycle, its phase consistency at times of stimulus onsets, and the angle at stimulus times to which it converges.

Note that (a) the focus was on the phase of the oscillator and not its amplitude; therefore, the system was simplified relative to a canonical coupled oscillator model (e.g., the one suggested by [43]) by fixing the amplitude; (b) to quantify the extent to which oscillators at different frequencies can be entrained to the stimulation streams, we used an array of oscillators with fixed periods instead of a single oscillator that can change its frequency. Therefore, the system was simplified relative to a canonical coupled oscillator model by removing the period correction term; (c) the model was implemented such that instead of receiving only the period and phase of the external stream as input (which would have only allowed rhythmic external streams, [44]), our model received a time series of stimulus energy (0 = no stimulus, 1 = full stimulus energy), such that stimulus bursts can have any desired SOA.

To estimate the ITPC values and phase angles expected in the experimental conditions, we numerically integrated this dynamical system using the Runge–Kutta method (fourth order). The frequency range of interest were 0.2 to 5 Hz (logarithmic scale), corresponding to interval lengths of 5,000 to 200 ms in 50 ms steps. The coupling parameter *c* was adjusted such that ITPC values in the Rhythmic condition at the stimulation frequency would be similar to those observed in the empirical data. For each participant and each experimental condition, we applied the following steps:

1. Generate the time series that represents a single trial in the current condition. The stimulation stream in this trial was represented as a train of impulses at the times of stimulus onsets. Specifically, we created a time series vector with a 1,000 Hz resolution (i.e., one value for every millisecond). This time series contained one at the time of stimulus onset in the actual trial and zero otherwise.
2. Determine the initial phase angle for the current trial. Phase angles were sampled without replacement from a uniform circular distribution of equally-spaced angles, the number of which was identical to the number of trials in the specific experimental condition (48 for the Rhythmic and Repeated-Interval conditions, 24 for the Random condition). The initial phase angle was identical to all oscillators stimulated by this trial, which guaranteed matching phase dispersion between frequencies and conditions.
3. Numerically integrate the system given the trial time series and initial phase angle (from stages 1 and 2, respectively), separately for each oscillator in the oscillator array, and register the angle at the time of the last stimulus in the stream (the target) for that oscillator.
4. Repeat steps 1–3 for each trial, in each experimental condition, to obtain a series of angles for each natural frequency (48 trials in the Rhythmic and Repeated-Interval, 24 in the Random).
5. Calculate the ITPC and the mean angle across trials, identically to how it was calculated for the EEG angle data, separately for each natural frequency.

Files of raw behavioral and EEG data, model simulation data, and cover information are deposited at the Dryad data repository (http://dx.doi.org/10.5061/dryad.5vb8h) [84].

## Supporting information

S1 Fig Controlling for spurious phase-RT correlation. Circular-linear correlation, calculated as in Fig 5A of the main text. Left: Correlation between RTs and delta phase at the time of the WS in the Rhythmic condition. Right: Correlation between RTs and delta phase at the time of the target in the Random condition. *p<0.05.
(TIF)

S1 Text Supporting methods. (DOCX)

## Abbreviations

BIC: bayesian information criterion
CI: confidence interval
CNA: climbing neuronal activity
CNV: contingent negative variation
EEG: electroencephalogram
ERP: event-related potential
ITPC: inter-trial phase coherence
PC: phase concentration
RT: response time
SOA: stimulus onset asynchrony
WS: warning signal
