# Supplementary figures and images for: Neural mechanisms of rhythm-based temporal prediction: Delta phase-locking reflects temporal predictability but not rhythmic entrainment
Source: PLoS Biol. 2017 Feb 10;15(2):e2001665. doi: 10.1371/journal.pbio.2001665 (PMC5302287; doi:10.1371/journal.pbio.2001665)

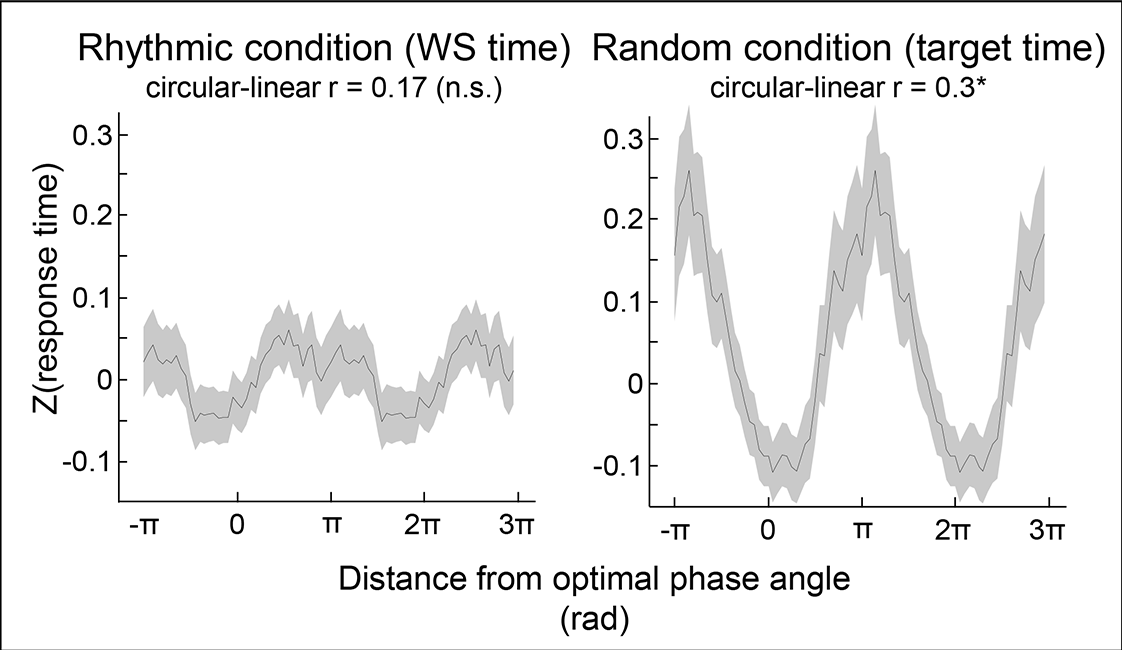

Supplement: S1 Fig — Circular-linear correlation, calculated as in Fig 5A of the main text. Left: Correlation between RTs and delta phase at the time of the WS in the Rhythmic condition. Right: Correlation between RTs and delta phase at the time of the target in the Random condition. *p<0.05. (TIF) [file pbio.2001665.s001.tif]
