# Supplementary material for: Neural mechanisms of rhythm-based temporal prediction: Delta phase-locking reflects temporal predictability but not rhythmic entrainment
Source: PLoS Biol. 2017 Feb 10;15(2):e2001665. doi: 10.1371/journal.pbio.2001665 (PMC5302287; doi:10.1371/journal.pbio.2001665)
Supplement: S1 Text — (DOCX) [file pbio.2001665.s002.docx]

### Jackknife estimation

To compare the parameters of the CNV models and the latency of the P3 between experimental conditions we used the jackknifing procedure [4-6], which allows statistical inference across subjects when reliable estimation of the dependent variable is difficult in single subjects (this is often the case for latency parameters). Instead, the parameters are estimated from n group averages (n = number of subjects) where in each average one of the subjects is omitted. The resulting values can then be used for statistical testing. However, appropriate corrections for variance shrinkage due to averaging should be applied, to maintain accurate rates of type I error [4, 5].

### Linear mixed-effect regression analysis

Observing an association between performance (here RT) and phase, such that performance is superior in specific phase ranges (optimal phases), is usually conceived as evidence that EEG oscillations reflect fluctuations in neural sensitivity. In single participants, this association can be tested by calculating the circular-linear correlation between phase (circular) and performance (linear). However, for group-level analysis, testing these values in a second level analysis (i.e. across subject) is problematic as their expected value under lack of association is not 0 (because of the circularity, the circular-linear correlation is a directionless statistic that varies between 0 and 1). One alternative approach is to pool single trials from all participants, while using a standardized measure for performance (e.g. accuracy or within-participant standardized RT) to eliminate inter-individual differences. However, this approach would still underestimate the strength of association if each participant had a different optimal phase. We propose that the appropriate analytic approach in this case is a multi-level regression technique known as linear mixed effect regression.

Linear mixed-effect regression is a technique that can handle designs with several random factors [7- 9]. It can be used to test the association of a predictor of interest (here, delta phase) with an outcome variable (here RT), across one random factor (here trials), when the latter is nested within another, second level, random factor (here subjects). In such situations, as in each subject the predictor and the outcome variables may have different ranges and different relationships, the estimates of the overall association between the predictor and the outcome can be distorted. The solution to this is fitting each subject with unique regression equations that have different intercepts and slopes for the predictor-outcome association. Consequently, the model accounts for four sources of variance in the outcome variable: variance explained by the predictor unvaryingly in all subjects (the fixed effect); variance explained by inter-individual differences in intercept between subjects (the random intercepts); variance that is explained by the predictors, but with a different slope in each subjects (the by-participant random slopes), and residual variance. The model is fitted to the data using maximum-likelihood estimates, and an effect of interest can be tested by conducting likelihood ratio tests of the full model against the model with all effects other than the effect in question, reporting a $\chi^{2}$-test statistic.

In the case of predicting RT from phase, the raw phase cannot be used as a predictor in a linear model due to its circularity. However, in a single participant the circular-linear correlation is obtained by taking the square root of the R^2^ of a linear regression model in which the linear variable is predicted from the sine and cosine of the circular variable. As the sine and cosine of the phase are not circular, they can be used as predictors in a linear model. Thus, in the linear mixed-effect model we followed this definition of the circular-linear correlation and used the sine and cosine of the phase as a set of predictors which jointly express the entire variance of the phase. The fixed effect of this set reflects dependence of RT on phase across participants, and the random slope effect of this set reflects the presence of subject-specific idiosyncratic optimal phases. This analysis was performed in R environment [10], using the lme4 package [11].

To visualize the association across all subjects, behavioral performance (RT) is presented as function of delta phase, averaged across all trials of all participants (Figure 5A). To allow meaningful averaging, RTs were standardized within participant. Furthermore, delta phases were aligned such that 0 is the optimal phase of each participant (estimated by averaging the fastest 33% trials for each participant), so that phase values on the x-axis are distance from the optimal phase. Note the cyclic dependence of RT on the phase.

### Permutation-based ANOVA

We used a permutation-based analysis-of-variance (pbANOVA) to assess modulations of inter-trial phase coherence (ITPC) by experimental conditions. pbANOVA is called for to analyze experimental designs with orthogonal manipulation of fixed factors in which the measured variable does not conform to the distributional assumptions necessary for traditional parametric ANOVA [12]. In this analysis, null distributions for the main effect of each factor and interaction are created using 10000 surrogate datasets, in each of which the data of a random subset of participants is permuted. In each iteration, the surrogate data is analyzed using a standard ANOVA and the F value of the relevant effect is registered. The effect in the original data is considered significant only if the F value of a standard ANOVA of this effect is larger than 95% of the values in the null distribution. For main effects, the permutations are conducted such that the raw values of the factor of interest are permuted within the levels of the other factor (termed 'restricted' permuting). For the interaction effect, the permutations are not conducted on the raw data but on a dataset that is generated by subtracting the contribution of the main effects from the raw data. The resulting dataset (termed 'reduced' dataset) includes only the interaction terms and their random errors. On this reduced dataset the permutations are conducted without being limited to levels of specific factors (termed 'unrestricted' permutations, see [12]).

### Controlling for spurious phase-RT correlation

Figure 5A demonstrates a circular-linear correlation between delta phase and RT, an essential pre-requisite for entrainment theories. To accumulate across subjects that may have different optimal phase (i.e. phase in which the RT is shortest), we aligned all subjects to an optimal phase of 0. However, this alignment procedure could in theory lead to seeming dependence between phase and RT even when there is none. When there is no true association between phase and RT, the phase of the fastest trials is random. However, as phase is a circular variable, the average of these random values does not ‘cancel out’, and subtracting it from all phase values would lead, by definition, to the phase of 0 having faster RTs than other phases. Hence, we had to find the baseline correlation that is expected when there is minimal true relationship between an event and the RT. For this purpose we used the time of the WS in the rhythmic condition, which is a well-defined event that is far enough from the response to assume minimal association with RT. Note that this choice is actually a highly conservative control, as the WS is a stimulus which could cause phase resetting, and it is embedded within a rhythmic sequence and could thus be actually phase aligned with the EEG (under the assumption of entrainment). The results of this control analysis are presented in Figure S1. Notably, although visually it seems like there is some association between delta phase in WS time and RT (left panel), this association is much weaker than what was observed in the Random condition (presented again for comparison in the right panel), and is not statistically significant. This finding reassures the assertion that the circular-linear association found in the Random condition does not reflect spurious correlation.

### Non-Informative condition

Prior to the experimental blocks, participants performed 3 blocks of a Non-Informative condition as replication of our previous study. In this condition the preparatory sequence is rhythmic as in the Rhythmic condition, but the target SOA is jittered around one of the two fixed SOAs, as in the Random condition. Thus, the rhythm is non-predictive of the target SOA, making it suitable to test the unintentional effects of exposure to rhythms [1-3]. The results of this condition are not reported in the current manuscript as most of them were described in our previous study (response speed, CNV, alpha-band activity and P3 in this situation, as well as direct comparison to a Rhythmic condition, see [1]). We do not compare this condition to the other conditions for several reasons. First, the delta phase analysis was not conducted in the Non-Informative condition because of the opposite effects of the putative entrainment due to the rhythm, promoting phase concentration on the one hand, and to the jittered target SOA dispersing the phase on the other hand. Specifically, had we observed low ITPC values in the non-informative condition, it would have been unclear whether there was no entrainment by the non-predictive rhythm, or whether there was entrainment, but as targets appeared in different SOAs the phase at target time was less consistent. Second, in the additional analyses the Non-Informative condition cannot be directly compared to the Repeated-Interval condition as they are different in two dimensions, the level of intentionality and of rhythmicity. As a result, it would have been unclear to what factor any differences between conditions should be attributed.

# References

1. Breska A, Deouell LY. (2014) Automatic bias of temporal expectations following temporally regular input independently of high-level temporal expectation. *J Cog Neurosci, 26*:1555-1571.
2. Sanabria D, Capizzi M, Correa A. (2011) Rhythms that speed you up. J Exp Psychol Hum Percept Perform, 37:236–244.
3. Jones MR, Moynihan H, MacKenzie N, Puente J. (2002) Temporal aspects of stimulus-driven attending in dynamic arrays. Psychol Sci, 13:313–319.
4. Miller J, Patterson T, Ulrich R. (1998) Jackknife­based method for measuring LRP onset latency differences. Psychophysiology, 35, 99-115.
5. Ulrich R, Miller J. (2001) Using the jackknife-based scoring method for measuring LRP onset effects in factorial designs. Psychophysiology, 38, 816-827.
6. Kiesel A, Miller J, Jolicoeur P, Brisson B. (2008) Measurement of ERP latency differences: A comparison of single-participant and jackknife-based scoring methods. Psychophysiology, 45, 250-274.
7. Baayen RH. (2008) Analyzing linguistic data: A practical introduction to statistics using R. Cambridge: Cambridge University Press.
8. Baayen RH, Davidson DJ, Bates DM. (2008) Mixed-effects modeling with crossed random effects for subjects and items. J Mem Lang, 59, 390–412.
9. Gelman A, Hill J. (2007) Data Analysis Using Regression and Multilevel / Hierarchical Models. Cambridge: Cambridge University Press.
10. R Core Team. (2012) R: A language and environment for statistical computing. R Foundation for Statistical Computing, Vienna, Austria.
11. Bates DM, Maechler M, Bolker B. (2012) lme4: Linear mixed-effects models using S4 classes. R package version 0.999999-0.
12. Anderson MJ, Ter Braak CJF. (2003) Permutation tests for multi-factorial analysis of variance. J Stat Comput Simul, 73, 85-113.
